# Complete Genome Sequences of Four Toxigenic *Clostridium difficile* Clinical Isolates from Patients of the Lower Hudson Valley, New York, USA

**DOI:** 10.1128/genomeA.01537-17

**Published:** 2018-01-25

**Authors:** Changhong Yin, Donald S. Chen, Jian Zhuge, Donna McKenna, Joan Sagurton, Guiqing Wang, Weihua Huang, Nevenka Dimitrova, John T. Fallon

**Affiliations:** a Department of Pathology, New York Medical College, Valhalla, New York, USA; b Department of Infection Prevention and Control, Westchester Medical Center, Valhalla, New York, USA; c Department of Pathology and Clinical Laboratories, Westchester Medical Center, Valhalla, New York, USA; d Department of Microbiology, Orange Regional Medical Center, Middletown, New York, USA; e Philips Healthcare North America, Cambridge, Massachusetts, USA

## Abstract

Complete genome sequences of four toxigenic *Clostridium difficile* isolates from patients in the lower Hudson Valley, New York, USA, were achieved. These isolates represent four common sequence types (ST1, ST2, ST8, and ST42) belonging to two distinct phylogenetic clades. All isolates have a 4.0- to 4.2-Mb circular chromosome, and one carries a phage.

## GENOME ANNOUNCEMENT

*Clostridium* (*Clostridiodes*) *difficile* is a Gram-positive, spore-forming, anaerobic bacterium that causes antibiotic-associated diarrhea and life-threatening pseudomembranous colitis. The incidence of *C. difficile* infections (CDIs) has dramatically increased in the last decade. It is estimated that approximately 453,000 incident infections with approximately 29,000 deaths occurred in the United States in 2011 (1). The pathogenicity of *C. difficile* is closely linked to two major exotoxins (toxin A and toxin B) encoded by genes *tcdA* and *tcdB*, respectively, in a pathogenicity locus (PaLoc) (2, 3). The virulence of *C. difficile* can be enhanced by producing binary toxin (CDT) (4, 5). Genes encoding the binary toxin, *cdtA* and *cdtB*, together with a regulation gene *cdtR*, are located on the CDT locus (CdtLoc) (6).

Genomic sequence analysis provides insights into the genomic diversity, evolution, and transmission of *C. difficile* (7). However, among 17 *C. difficile* complete genomes publicly available to date in the GenBank database (https://www.ncbi.nlm.nih.gov/genome/genomes/535, accessed on 11 December 2017), only 3 (BI1, DH, and FDAARGOS_267) were from humans in the United States. In this study, we sequenced four *C. difficile* clinical isolates (W0003a, W0022a, W0023a, and R0104a) from patients of the lower Hudson Valley, New York, USA. These isolates were recovered from patients with symptomatic CDIs and represented the most common strains in this region. Total genomic DNA of each isolate was extracted with a Qiagen QIAamp genomic DNA kit. Whole-genome sequencing was performed on both the Pacific Bioscience (PacBio) RS II and Illumina MiSeq platforms. A sequencing library for the MiSeq platform was prepared using the Illumina Nextera XT DNA sample prep kit, and paired-end (2 × 150-bp) sequencing was performed. A library for the PacBio single-molecule real-time (SMRT) sequencing system was processed with the PacBio SMRTbell template preparation kit. Genome sequences were *de novo* assembled by PacBio SMRT analysis and/or the SPAdes assembler (8), polished by aligning and mapping the short reads generated from the MiSeq run, and visualized under the Integrative Genomics Viewer (9).

The genomes of W0003a, W0023a, and R0104a each contain a single circular chromosome of 4,075,361, 4,110,080, and 4,190,038 bp, respectively. W0022a consists of a circular chromosome of 4,188,456 bp and a circular phage of 31,888 bp. The sequence type (ST) of each *C. difficile* strain was determined by querying the whole-genome sequence to the public multilocus sequence typing (MLST) database (http://pubmlst.org/cdifficile): R0104a (ST1), W0022a (ST2), W0003a (ST8), and W0023a (ST42). The four isolates represent the most common strains recovered in this region. MLST-based phylogeny further designated these isolates into two distinct phylogenetic clades: R0104a as clade 2, and the others as clade 1.

Compared to the reference genome *C. difficile* 630 (GenBank accession number NC_009089), the genomes of all four toxigenic *C. difficile* isolates contain intact *tcdA* and *tcd*B genes in the PaLoc, of 7,133 and 7,101 bp, respectively. In addition, a complete CdtLoc, including intact binary toxin genes *cdtA* and *cdt*B and their regulatory *cdtR*, was identified in R0104a, whereas an incomplete CdtLoc, containing only the *cdtR* gene, was observed in isolates W0003a, W0022a, and W0023a (5, 10).

### Accession number(s).

Complete genome sequences of the four isolates reported here have been deposited in GenBank under the accession numbers CP025044 to CP025047.
